# Immuno-histopathologic evaluation of mineralized plasmatic matrix in the management of horizontal ridge defects in a canine model (a split-mouth comparative study)

**DOI:** 10.1007/s10266-021-00684-3

**Published:** 2022-01-05

**Authors:** Souzy Kamal Anwar, Hend Mohamed Abdel Hamid

**Affiliations:** 1 Oral Medicine, Periodontology, Oral Diagnosis and Radiology Department, Faculty of Dentistry, Alexandria University, Champolion St. Azarita, Alexandria, 21521 Egypt; 2 Oral Pathology Department, Faculty of Dentistry, Alexandria University, Champolion St. Azarita, Alexandria, 21521 Egypt

**Keywords:** Bone regeneration, Collagen, Critical size defect, Osteopontin, Platelet-rich fibrin

## Abstract

Our research aimed to investigate the effect of combining biphasic calcium phosphate (BCP) alloplast with mineralized plasmatic matrix (MPM) as compared with platelet-rich fibrin (PRF) on the quality and quantity of bone formation and maturation at surgically created horizontal critical-sized ridge defects (HRDs) in a canine model. We used a split-mouth design using the third and fourth mandibular premolars of the mongrel dogs. Twelve defects on the left side (experimental group, I) were managed with MPM composite mixed with BCP alloplast, MPM compact layer. On the right side (control group, II), another 12 defects were managed with PRF mixed with BCP alloplast, followed by the application of PRF compact strips. Finally, both were covered by a collagen membrane. Dogs were euthanized at 4, 8, and 12 weeks, and the studied defects were processed to evaluate treatment outcome, including mean percentage of bone surface area, collagen percentage, and osteopontin (OPN) immunoreaction. Our results revealed that the mean percentage of bone surface area was significantly increased in the experimental group treated with MPM at all time intervals as compared with the PRF group. Decreased collagen percentage and increased OPN immunoreactivity showed significant results in the MPM group as compared with PRF at 4 and 8 weeks postoperatively, respectively. In conclusion**,** MPM accelerates the formation of superior new bone quality when used in the treatment of HRDs.

## Introduction

The esthetic profile for any restoration depends mainly on marginal gingiva and interdental papillae support, which achieved by adequate volume and height of the alveolar ridge. Moreover, this support is mandatory for restoration’s function [1–3]. Severe jaws atrophy presents a difficult challenge in mouth rehabilitation, when being augmented with graft materials. Since reduction in bone volume leads to restriction in the total vital bone area which will be in contact with the graft material. Meanwhile, the success of graft material relies on the existence of vital bone facilitating angiogenic spread into grafted volume, transporting cells, growth factors, nutrients, and oxygen [4].

Improving regenerated bone quantity as well as quality is the ultimate goal for successful periodontal therapy which includes the use of numerous biologic mediators [5]. Bone grafting methods currently face several limitations and require new, alternative techniques for repairing bone defects [6]. Hence, trials have been conducted not only to minimize donor-sites-related surgical complications with autografts but also to improve treatment outcomes. These trials used novel tissue-engineered techniques with the aim of improving the quality of the regenerated bone in critical-sized defects [7–10].

In attempts to surpass bone regeneration capacity, numerous growth factors alone or with grafting materials have been evaluated for ridge augmentation in different animal models. Platelets-rich plasma (PRP) was the first generation used in reconstructive periodontal therapy [11]. However, PRP exhibited weak regeneration potentials with regard to hard-tissue formation, with its therapeutic application rendered extremely difficult by several technical-sensitive steps [12].

To defeat PRP drawbacks, Choukroun et al. [13] introduced platelet-rich fibrin (PRF), the second generation of platelet concentrates. PRF is a completely autologous fibrin-rich gel manufactured from the patient’s own venous blood. The chief advantage for PRF was its simplicity of synthesis protocol and it does not need any biochemical or chemical supplement as bovine thrombin or calcium chloride to reach the gel state. Additionally, the PRF gel stimulates several growth factor liberations, which aids in the acceleration of new bone formation as well as soft tissue healing [14, 15].

Despite all these advantages, the new ridge contour volume created by PRP or PRF often collapse by the action of chewing forces along with flap muscles’ movements [16]. This is due to PRF nature, as it is not homogenized smoothly with bone substitute crystals [16]. Moreover, conventional extraction techniques do not allow the mixture of PRF coagulum and inorganic compound to form a single homogeneous product as it involves sequential materials addition [16].

To overcome such challenges, Périssé [16] introduced the technique of mineralized plasmatic matrix (MPM). The clinical advantage of MPM technique is that refines the quality of the bone graft/PRF mixture creating a stable, homogeneous, single-moldable compound with more relevant properties rather than the establishment of a heterogeneous compound formed of bone and PRF [16]. This homogeneity of MPM offers an easier clinical operation for handling fillers into the defect, with additional osteoinductive biological properties [16]. Another unique feature of MPM is the ability to adhere to the bone surface after application, which further enhances its stability in the recipient bed [17, 18].

However, most of the studies involving MPM have been performed in vitro [16, 19, 20]. Meanwhile, in vivo studies that discriminate large animal models with critical-size clinical relevance defects are limited without histopathological assessment. Only one study was performed on sheep, but the methodology was not clear, and the author did not use critical-sized defects [20]. Recent study has shown that MPM technique is valuable and predictable in obtaining bone fill in the maxillary and mandibular sockets with residual crestal ridges deemed necessary for ridge preservation in implant therapy [21]. On the same hand, another has proved the effectiveness of MPM in the closure of the cleft defect and oro-nasal fistula [22].

Within this context, osteopontin (OPN) is a component of the mineralized extracellular matrix crucial for biomineralization seen in bone remodeling. The secreted OPN concentrated along the wound bone surface promoting adhesion novel sites for osteoblasts recruitment and subsequent differentiation in the interface between older bone and the newly formed one. This is highly important to initiate the early stages of bone mineralization by the cement lines bonding newer bone to older bone [23].

In our study, we aimed to investigate the effect of combining biphasic calcium phosphate (BCP) alloplast with mineralized plasmatic matrix (MPM) as compared with platelet-rich fibrin (PRF) on the quality and quantity of bone formation and maturation for surgically created horizontal critical-sized ridge defects in a canine model. The results of our study were based mainly on histoimmunoanalysis of extracted tissues. We used this analysis method because it is the only assessment that can ascertain the actual occurrence and the true extent of tissue regeneration in addition to viewing the quality and quantity of the reconstructed bone architecture [24, 25].

## Materials and methods

### Animal model

A split-mouth comparative experimental study design was conducted in accordance with the highest standards of ARRIVE guidelines under the approval of the Research Ethics Committee of the Faculty of Dentistry, Alexandria University for the conduct of research on experimental animals by the Faculty of Dentistry, Alexandria University (IRB No. 00010556-IORG 0008839). We used this study design to standardize all factors that could affect the regenerative procedure [26, 27].

Sample size was estimated assuming alpha error = 5% and study power = 80%, based on previous studies on the effect of BCP alloplast, MPM, and PRF on bone formation [28–30]. To detect an effect size of 1.32 (difference in bone formation between experimental and control groups), using two-tailed test, the required sample size was calculated using G*Power 3.1.9.4 sample size calculator [31] to be 11 defects per group, increased to be 12 defects to make up for the loss to follow-up. Therefore, a sample of six dogs was the enough required sample for this study, as the aim of the study was to demonstrate intervention efficacy in a single group utilizing a split-mouth study design (4 defects per dog) (number of groups = 2) (total number of defects = 24 defects). To act as a negative control, another group of three dogs (12 defects; 4 defects in each dog) was added in which the defects were created and left empty. Accordingly, the total sample size was nine dogs (12 defects per group) (number of groups = 3) (total sample size = 36 defects).

For this study, we selected nine healthy, adult, male mongrel dogs (*Canis familaris*), about 18–24 months of age and weighing approximately 9–14 kg. The dogs were supplied and housed by the City of Scientific Research and Technological Applications (SRTA-City). We chose a canine model instead of a rodent animal since skeletally larger animals are more suitable in studies targeting dental tissue healing [32].

The mandibular third (P3) and fourth (P4) premolars of the nine dogs were assigned for this study. The study included three groups, each included 12 surgically created horizontal, well-contained critical-sized ridge defects. In group I (MPM experimental group), 12 defects on the left side of 6 dogs (2 defects in each dog) were created and managed with GBR using MPM composite and covered with an MPM compact layer followed by the application of collagen membrane (COLLAGUIDE™ by BIOLAND, Korea). In group II (PRF control group), 12 defects on the right side of the same 6 dogs (2 defects in each dog) were created and managed with GBR using PRF cuts mixed with BCP alloplast (Genesis BCP™ by Dio-implant, Korea) and covered with PRF compact membrane followed by the application of collagen membrane. Regarding the six dogs of the experimental and control groups, two dogs (number of defects = 8; 4 for each group) were randomly allocated using computer-generated list of random numbers and euthanized as follows: two dogs at 4-weeks, another two after 8-weeks, and the remaining two after 12-weeks. In group III (negative control group), 12 defects were created and left empty in three dogs (4 defects in each dog, 2 defects on the right side and 2 on the left) that were euthanized at 12 weeks. That was done to cover the whole study period showing that such a defect will not regenerate spontaneously without adjunctive measures, allowing for an unbiased strategy for analysis of the obtained results [33, 34]. This was done in contrary to the study conducted by Cakir et al. [35] to confirm the critical-sized defect dimensions.

### MPM preparation

**Fig. 1:**
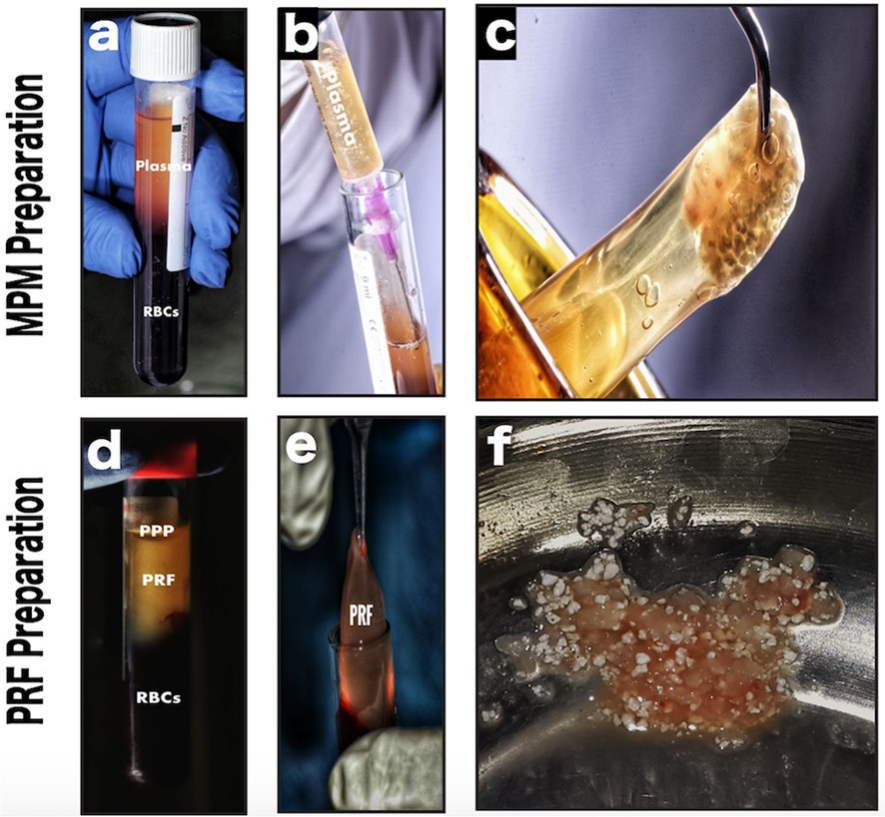
Clinical photographs showing MPM versus PRF preparation and their mixing with alloplast. **a** Plain vacuumed plastic tubes, Z no additives, used for MPM preparation containing the separated blood after centrifugation, into two layers: topmost layer of clear yellow plasma and red blood cells (RBCs) at the bottom. **b** Collection of the liquid yellow plasma using a syringe. **c** The resultant homogenous mixture of MPM composite. **d** Plain vacuumed glass tubes used for PRF preparation containing the separated blood after centrifugation, into three layers: topmost layer of platelet-poor plasma (PPP), platelet-rich fibrin (PRF) clot in the middle and RBCs at the bottom. **e** PRF clot separation. **f** The resultant heterogeneous mixture PRF cuts with alloplast

The MPM was prepared according to the stated protocol published by our group [35]. Briefly, we draw 20 ml venous blood from the animal into anticoagulant-free plastic blood collection tubes (VACUETTE® Z No Additive by Greiner Bio-One GmbH, Austria; 10 ml each) for centrifugation (15 min at 2500 rpm). This led to the separation of blood into red blood cells in the bottom and an upper layer containing clear yellow plasma rich in leukocytes, platelets, mesenchymal stem cells, and fibrinogen. Then, we collected the upper layer and mixed it with BCP to form a homogenous mixture as shown in Fig. 1a–c.

### PRF preparation

PRF was prepared according to Chokroun’s technique [37]. Briefly, we draw 20 ml of venous blood from the cephalic vein of each dog and immediately divided into two anticoagulant-free glass blood collection tubes (SUMBOW® Plain Glass Vacuum Tube, China; 10 ml each) and centrifuged for 10 min at 3000 rpm. The fibrin clot formed in the middle of the tube after centrifugation, was gently removed and cut into small pieces and mixed with the BCP to fill the two created defects. The other fibrin clot was used to obtain the PRF membrane. (Fig. 1d–f).

### Surgical procedure

**Fig. 2:**
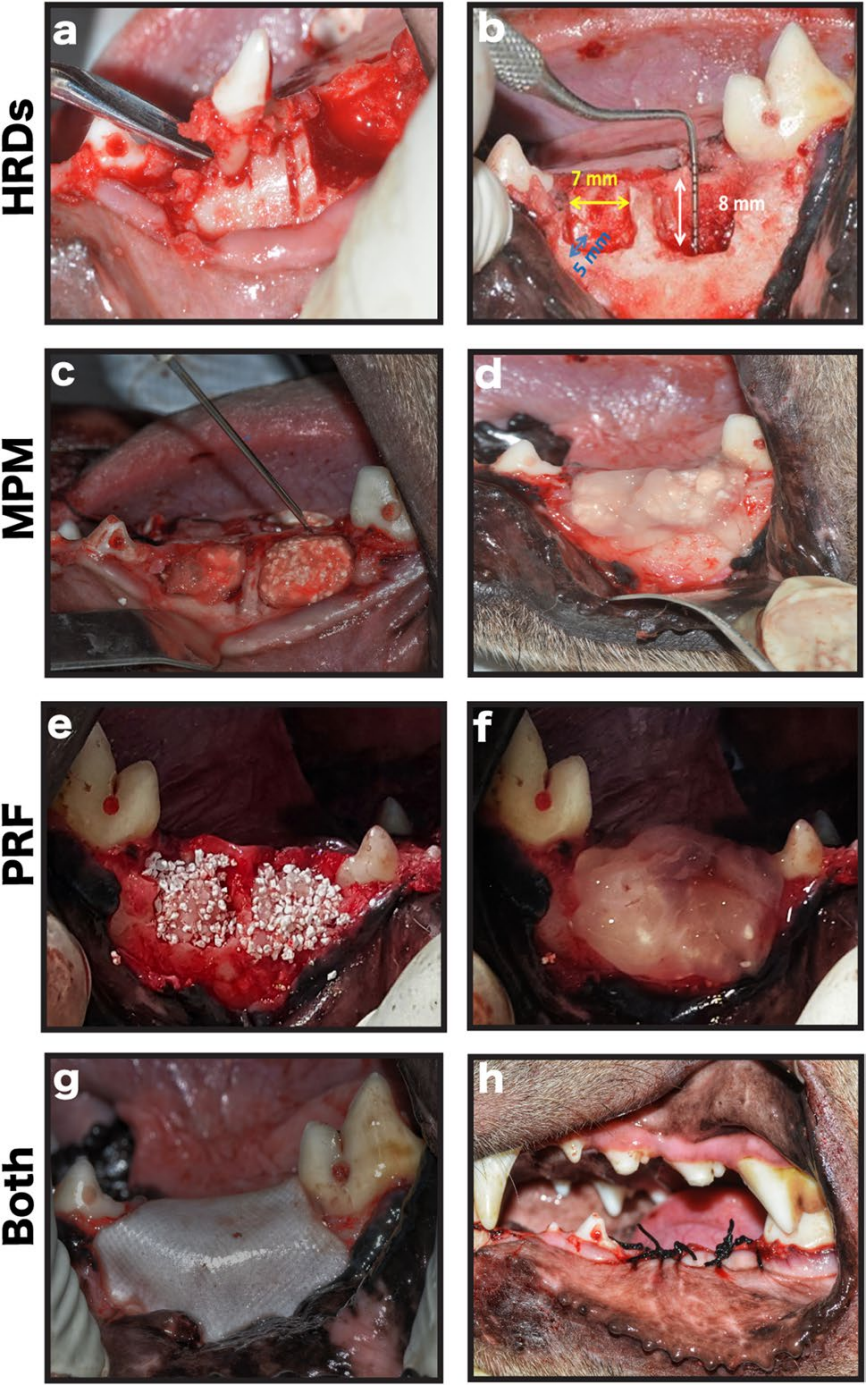
Clinical photographs showing the steps of the performed surgical procedure. **a** Teeth extraction. **b** The created two well-contained critical-sized horizontal ridge defects per jaw quadrant, each about 7-mm mesio-distally × 8-mm apico-coronally × 5-mm bucco-lingually. **c** Management of the study side with MPM composite and injection of part of the collected plasma over the formed MPM composite to form the MPM compact layer over it. **d** Defect covered with MPM compact layer. **e** Management of the control side with alloplast mixed with PRF cuts and serum exudates. **f** Defect covered with PRF membrane. **g** Collagen membrane placement to completely cover the defects on both sides. **h** The flap advancement to cover the membrane, suturing with simple interrupted sutures and reduction of the cusp tips of the teeth in the upper jaw opposite to the defect site

The animals were anesthetized via an intravenous injection of sodium thiopental (13 mg/kg). We performed sulcular incisions and reflected the buccal mucoperiosteal flaps at the regions of the mandibular third (*P*_3_) and fourth (*P*_4_) premolars. Then, we extracted premolar teeth. Following Cologne Classification of Alveolar Ridge Defects (CCARD); the defects dimensions after extraction were surgically extended mesiodistally, buccolingually and apicocoronally to meet the criteria of horizontal medium sized defect 4–8 mm, inside the ridge contour (H.2.i) [38]. Accordingly, two horizontal well-contained critical-sized ridge defects were surgically created per jaw quadrant, each about 7-mm mesiodistally × 8-mm apicocoronally × 5-mm buccolingually [33]. (Fig. 2a,b) Acute-type surgical defects were used because these defects are well characterized, standardized wound models that are reproducible, allowing for an unbiased and appropriate strategy for analysis of the obtained results [34, 39].

For the experimental group (left side), we treated the defects with MPM composite covered with MPM compact layer. On the right side, the control group, we fill the defects with the mixture of the BCP with serum exudates and PRF cuts covered with PRF membrane. Then, we covered all the defects with collagen membrane and advanced the flaps to their original positions and sutured using simple interrupted 2–0 silk sutures (Ethicon silk suture, Johnson & Johnson, USA; Fig. 2c–h).

On the first postoperative day, animals were injected with ampicillin (Eipico Co., 10th of Ramadan City, Egypt; 1 g) intramuscularly and then received ampicillin administered in their food for 7 days. They were also injected intravenously with a nonsteroidal anti-inflammatory drug (Meloxicam DELTA PHARMA Factory-Industrial Zone B4,10th of Ramadan City, Egypt) on the first day. Dogs were placed on a soft diet throughout the postoperative period to reduce the possibility of local trauma to the site of operation. Sutures were removed after 10 days. If signs of surgical failure were noticed as wound dehiscence or infection, the defects were retreated and excluded from the study. Dogs were euthanized via an intravenous overdose injection of concentrated thiopental sodium.

### Histologic evaluation

After the animals were euthanized, jaw segments with the operated teeth were dissected and fixed in 10% buffered neutral formalin for 4 days. Segments were then decalcified in multiple baths of 10% trichloroacetic acid. After decalcification, the specimens were immersed in paraffin blocks to obtain semi serial (5-µm) thick histologic sections in the buccolingual direction through the entire mesiodistal plane of the premolars using a rotatory microtome. Finally, tissue sections were stained with hematoxylin and eosin (H&E) for general evaluation of tissue healing and cell activity and to show the sequential changes in regenerative features during osteogenesis between MPM and PRF [39]. To accomplish this, we examined H&E stained the sections at three follow-up intervals: 4, 8, and 12 weeks postoperatively. This was done to show the quality of newly formed bone and the distinction between immature woven bone and mature lamellar bone. In addition, Gomori trichrome stain was used to examine the new bone formation and collagen organization [40].

### Histomorphometric analysis

Histologic sections were analyzed quantitatively using ImageJ 1.46 r software (all obtained a magnification × 100) [41]. The mean percentage (%) of the newly formed bone surface area in the defect parameter was measured for the three groups at the three different observation periods. Five images were used for each section, and two different pathologists blindly evaluated all measurements, and the means were recorded.

### Quantitative analysis for percentage of collagen fibers

Tissue sections were stained by Masson’s trichrome, and the stained sections were quantitatively and blindly analyzed using ImageJ 1.46 r software (all obtained at a magnification × 100) [41]. Five different microscopic fields were used for each specimen to quantify the collagen percentage.

### Immunohistochemistry staining

Tissue sections were stained using osteopontin (OPN; Thermo Scientific, Cat. No. RB-9097-R7, USA) at a dilution ratio of 1:50 of rabbit polyclonal anti-OPN antibody. This was done to investigate early events in extracellular matrix mineralization. Immunohistochemical staining was performed using the labeled streptavidin–biotin complex method [42]. The immunoreactivity of the tissue sections was examined blindly by two different pathologists in five randomly selected microscopic fields at 400 × magnification to determine the intensity as the mean area percentage and were analyzed quantitatively using ImageJ 1.46 r software. Positive cells were counted in five different microscopic fields that demonstrated more intense staining.

### Statistical analysis

Data analysis was performed using IBM SPSS software package version 22.0 (IBM Corp, Armonk, NY) [43]. Normality was checked for all variables using descriptive statistics, plots (histogram and boxplots), and Shapiro Wilk normality test. All variables showed normal distribution, so means and standard deviation (SD) were calculated, and parametric tests were used. Paired *t* test was used for comparisons between MPM and PRF groups, repeated-measures analysis of variance for comparisons between more than two periods or stages and post hoc tests (least significant difference) for pairwise comparisons.

## Results

### Clinical evaluation

The animals tolerated the surgical procedures well and recovered normally. Furthermore, all dogs survived the entire study period. There were no postoperative adverse reactions, such as allergies or infection. Moreover, neither exposure of the collagen membrane nor tissue dehiscence was observed.

### Histologic evaluation

**Fig. 3:**
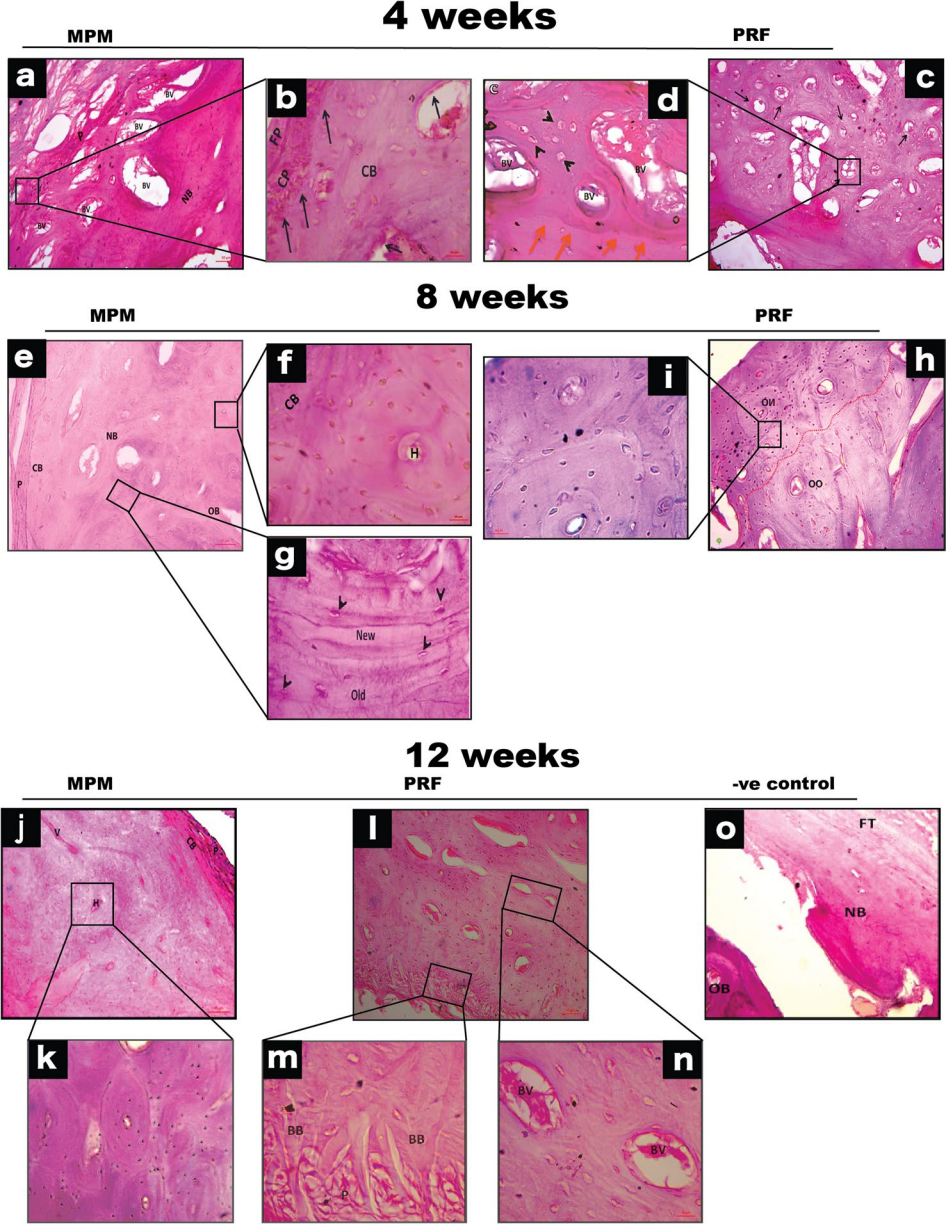
Histological evaluation of MPM and PRF in management of HRDs for 4-, 8- and 12-weeks. **a**, **b** Photomicrograph for MPM treated group after 4-weeks showing thick new bone formation (NB) around blood vessels (BV), covered by periosteum (P). **c**, **d** Photomicrograph for PRF group at 4-weeks showing high connective tissue stroma replaced by early developed immature bone with numerous osteocytes (arrowhead) and the line of demarcation between old and new bone. **e–g** Specimens for MPM at 8-weeks revealing circumferential bone (CB), covered by thick periosteum (P), superficial to the underlying new osteons of compact bone (NB) deposited over old ones (OB). **h,i** Tissue section for PRF group showing small new osteons (NO) deposited over large old ones (OO). Also, show the connection between old and new bone (red dotted line). **j**, **k** 12-weeks postoperatively, MPM managed group reveal well-organized, dense compact bone masses formed of numerous mature osteons equal in size, under circumferential bone (CB) that is covered by thick periosteum (P). **l** Photomicrograph for PRF at 12-weeks’ time point showing highly cellular newly formed osteon under the periosteum (P) which are immature and not equal in size with haphazard cellular organization around large blood vessels (BV). **m** demonstrates the connection between bundle bone (BB) and the overlying periosteum (P) **n** reveal large-sized blood vessels (BV) surrounded by numerous large osteocytes with the haphazard cellular organization. **o** Photomicrograph for the negative control group 12-weeks postoperatively, with new bone (NB) consisted of branching slender, immature trabeculae that are widely spaced over old bone (OB) and covered by thick fibrous tissue (FT). (All sections are stained with H&E × 100 and insets are H&E × 400)

We examined the tissue sections to show the sequential changes in regenerative features during osteogenesis between MPM and PRF. Four weeks postoperatively, tissue sections reveal the difference between dogs treated with MPM versus PRF. The defects treated with MPM appeared to be replaced by thick, dense bone trabeculae covered by periosteum and thick collagen membrane on the surface. In the PRF group the new bone appears as irregular, immature, unorganized woven bone starting to take the arrangement of primary osteons with multiple dilated blood vessels. (Fig. 3a–d).

Bone maturation continued during the 8-weeks follow-up, with a mature dense bone trabecula forming merged homogenous masses that filled almost all defects in the MPM group blending with the preexisting bone. However, in the PRF group, alternating bright and dark staining bone trabeculae were seen. The stain variation reflected the influence of the time factor on the amount and quality of the more mineralized forming bone, replacing areas of fibrous tissue. (Fig. 3e–i).

Throughout the entire defect, bone formation was evident and was made of well-organized, dense, compact bone masses formed of numerous equal-sized osteons in the MPM group 12-weeks postoperatively. On the other hand, the osteons in the PRF group were highly cellular, immature, and unequal in size, with a haphazard cellular organization around the large blood vessels (Fig. 3j–n).

In the current study, the negative control defect was left for 12-weeks to demonstrate whether any bone formation would spontaneously fill the critical defect space. The defect was infiltrated by large marrow spaces, with some granulation tissue in between with a limited amount of bone formation underlying the thick layer of fibrous tissue (Fig. 3o).

### Histomorphometric analysis

**Fig. 4:**
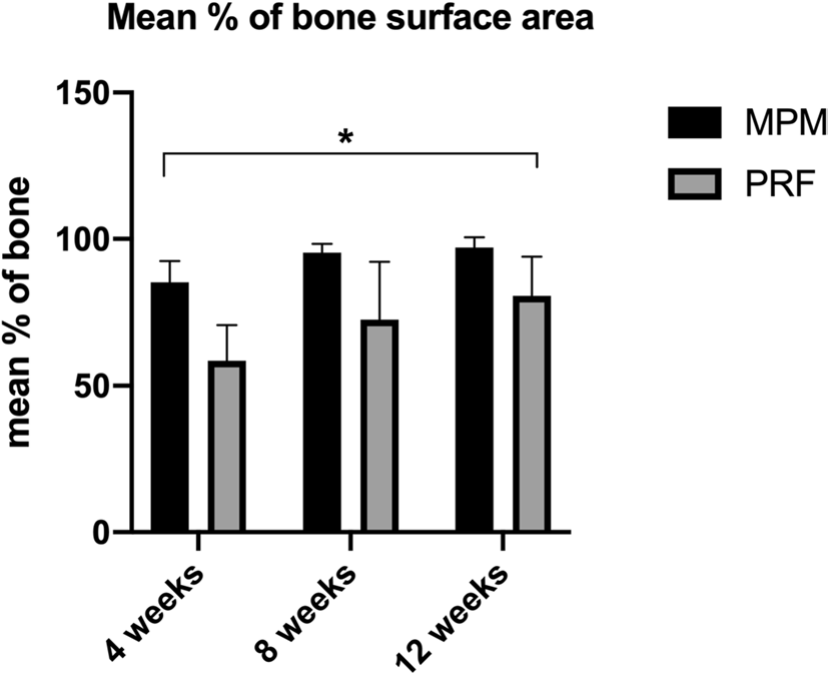
A representative graph showing the difference in mean percentage of bone surface area in all groups. A statistically significant increase in the mean percentage of bone surface area in MPM compared with PRF group was seen at all study period (4-,8-, and 12-weeks) (*P* < 0.05) denote by asterisks

The MPM group demonstrated a statistically greater mean percentage of bone surface area at 4, 8, and 12 weeks postoperatively (85.24 ± 7.16, 95.28 ± 3.06, and 97.07 ± 3.47, respectively) than the PRF group (58.52 ± 12.08, 72.44 ± 19.83, and 80.54 ± 13.28, respectively; *P* < 0.001, 0.004, and 0.002, respectively; Fig. 4).

### Quantitative analysis of the percentage of collagen fibers

**Fig. 5:**
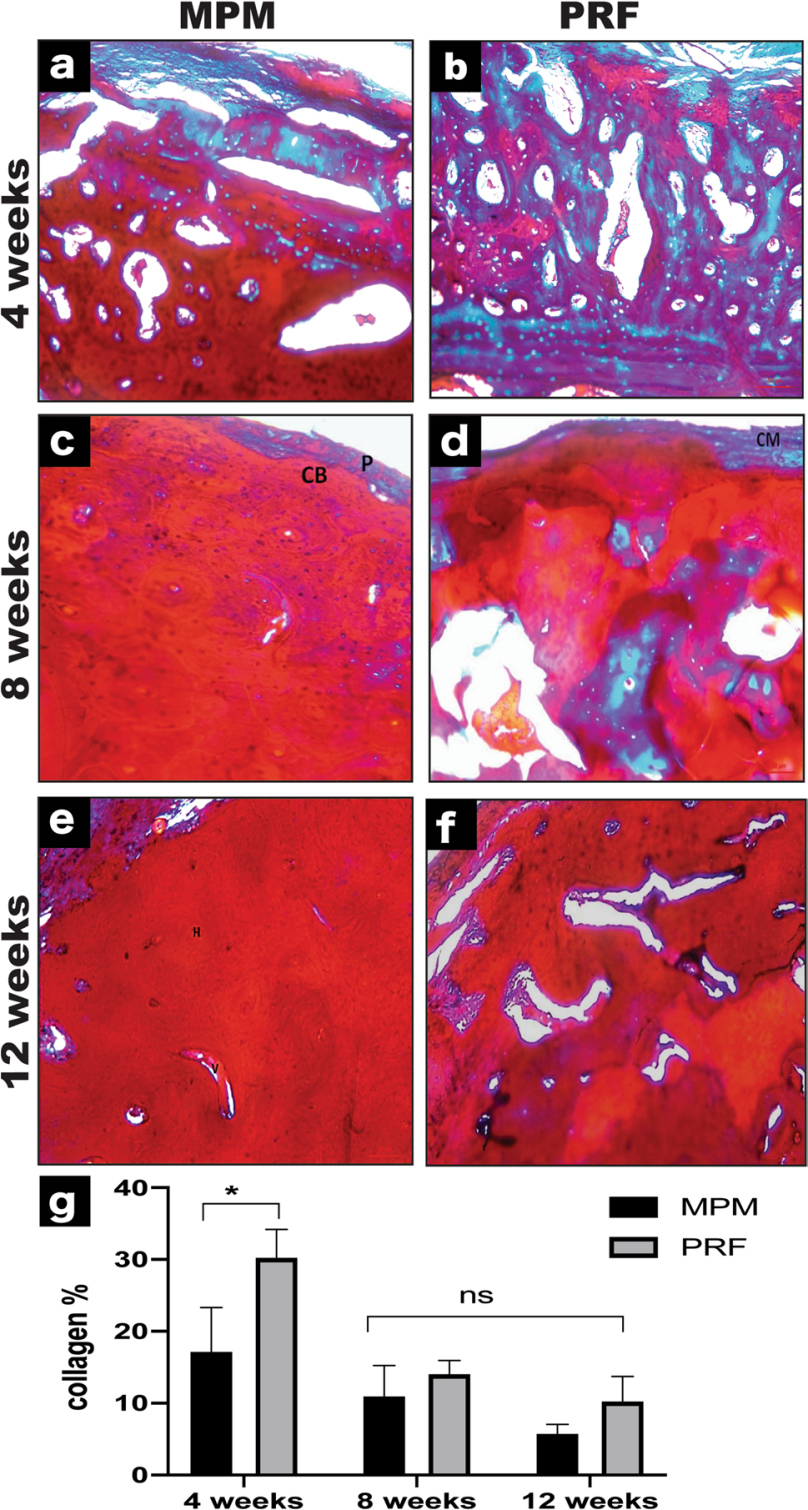
Histological evaluation of MPM and PRF using Masson's trichrome staining for collagen quantification. **a**, **b** 4 weeks’ time interval for MPM and PRF, respectively. **c** Specimens for MPM at 8-weeks revealing circumferential bone (CB), covered by thick periosteum (P). **d** Tissue section for PRF group at 8 weeks showing collagen membrane (CM). **e**, **f** 12 weeks’ time interval for MPM and PRF, respectively. **g** A representative graph showing the difference in collagen percentage between MPM and PRF across all study periods. A statistically significant decrease in bone matrix collagen % in MPM compared to PRF group was seen only at 4-weeks interval (*P* < 0.05) denote by an asterisk. (All sections are stained with Masson's trichrome × 100)

Upon quantifying the percentage of collagen, we found that defects treated with MPM induced regenerative bone formation after 4-weeks, with an average 17.11% ± 6.23% of collagen fibers compared with 30.17% ± 3.99% in those treated with PRF (*P* = 0.04). At the 8-weeks time point, the MPM and control groups showed a decrease in bone matrix collagen fibers average percentage, 10.86% ± 4.34% and 14.04% ± 1.96%, respectively, with no statistical significance (*P* = 0.39). The same results were noted during the 12-weeks in which a decrease in collagen percentage continued to occur, reaching 5.70 ± 1.34 and 10.17 ± 3.52 in the MPM and PRF groups, respectively (*P* = 0.25). Moreover, the decrease in collagen percentage in the MPM group that occurred during bone maturation across the three-time intervals showed no statistical significance (*P* = 0.11). Meanwhile, there was a significant difference in PRF collagen percentage between the 4- and 8-weeks time point (30.2% ± 4% and 14% ± 2%, respectively; *P* = 0.045; Fig. 5a–g).

### OPN immunoreaction

**Fig. 6:**
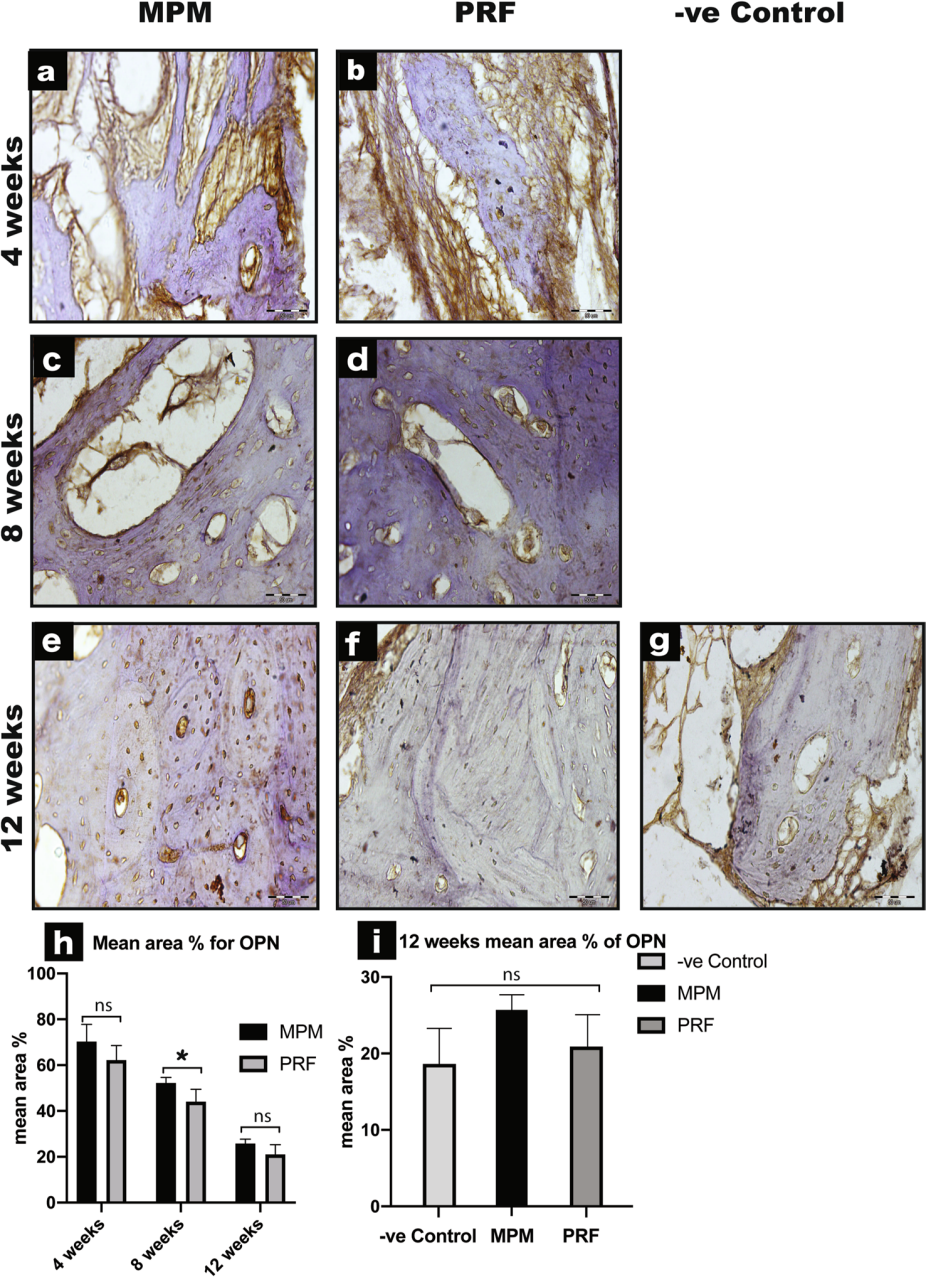
Immunohistochemical expression of OPN from treated dogs with MPM and PRF across all time intervals. **a**, **b** Photomicrographs showing changes in OPN immune-positivity at 4-weeks’ time point OPN with strong positive immune expression in the extracellular matrix in both MPM and PRF with no significant difference. **c**, **d** Decrease in OPN immune-staining is seen at 8-weeks in both MPM and PRF. **e**, **f** Further decrease in OPN staining is noted in both MPM and PRF treated groups at 12-weeks’ interval with linear deposition of OPN immunoreaction across the cement lines in MPM while, less homogeneously dispersed OPN immune positivity throughout the osteon was noted in the PRF group. **g** Photograph for the negative control group with reduced OPN immunoreaction seen only in the scanty bone marrow. **h** A representative graph showing the difference in mean area percentage of OPN immunoexpression between MPM and PRF across all study periods with a statistical significance at 8-weeks interval denote by an asterisk (*P* < 0.05). **i** A representative graph illustrating 12-weeks mean area percent for MPM, PRF and negative control groups showing almost the same results for all groups with no significant difference. (All sections are stained with OPN × 400)

After assessing the bone surface area, we analyzed the immunoreaction of OPN as a bone-specific protein found in the extracellular matrix. We observed high immune positivity to OPN in the MPM group as compared with the PRF-treated group (MPM 70.32 ± 7.52, 52.24 ± 2.41, 25.75 ± 1.94, respectively; PRF 62.25 ± 6.38, 43.97 ± 5.42, 20.96 ± 4.32, respectively) throughout the three-time point intervals. However, a statistically significant difference between MPM and PRF was noted only at the 8-weeks interval (*P* = 0.03). At the 12-weeks interval, a linear deposition of OPN immunoreaction was observed across the cement lines at the periphery of the Haversian system in the MPM group. On the other hand, a less homogeneously dispersed OPN immune positivity throughout the osteon was noted in the PRF group. Moreover, when we compared the reaction during the 12-weeks to the negative control group, we noted a decrease in the OPN reaction that was not statistically significant (*P* = 0.18; Fig. 6a–i).

## Discussion

In this study, we investigated the effects of PRF and MPM using the GBR approach with BCP alloplast in the management of surgically created horizontal critical-sized ridge defects. We selected a canine model based it is more suitable for studying dental hard-tissue healing and because of the limited number of preclinical studies performed on large animals [32]. Moreover, we focused on histoimmunoanalysis of the tissues, which is considered more accurate for determining the true extent of regeneration as well as the bone type and degree of bone maturation compared with clinical and radiographic examinations only [25].

One of our study design limitations is that we separated the negative control group, although the study design was split mouth. We chose this design based on the ethical considerations of the Research Ethics Committee in our institution. The aim of adding a negative control group was to prove that such a defect will not regenerate spontaneously without adjunctive measures, allowing for an unbiased strategy for analysis of the obtained results [33, 34]. Accordingly, we separated the control group to be euthanized all at the end of the study to cover the whole study period and prove that these defects dimension’s is critical-sized.

In our study, following the principles of GBR, platelet concentrate forms were mixed with BCP alloplast particles. To standardize the study approach, the GBR technique was used in both groups, so any histological changes observed in either group will be attributed mainly to the role of the applied material.

We chose BCP ceramics to serve as the scaffolding matrix material, because this mixture of 40% *β*-tricalcium phosphate (*β*-TCP) + 60% hydroxyapatite (HA) possesses the reactivity of *β*-TCP and the stability of HA, providing more bioactivity. Furthermore, they have a controlled, slow degradation rate that aids in space maintenance of the defect during the study period [44–49]. Moreover, in vitro studies demonstrated that BCP stimulates osteogenic differentiation of human mesenchymal stem cells [50]. Consequently, its use in the current study could help in the osteogenic differentiation of mesenchymal stem cell extracts in the prepared platelet concentrates.

The most striking clinical observation and advantage of PRF and MPM materials revealed in this study was its adhesive property. This property keeps the particles of alloplast together, attaching them tightly to the walls of the defect; in addition, the membrane was adherent to the collagen membrane. This adherence is thought to provide strong stabilization of the membrane, which is a prerequisite for successful GBR procedures for preventing the downward growth of the epithelium and space maintenance.

Following the mixing of MPM, a stable, single pliable homogenous product resulted, which made handling the filler easier, which is an extra advantage of MPM over the PRF technique. This property could be linked to the nature of MPM as a biologically solidified bone graft entrapped in the fibrin network. It does not scatter the alloplast even upon being shaken with pliers, because bone substitute particles are strongly interconnected with each other by the fibrin network [16, 20]. Consequently, this helped in evaluating the efficacy of the adhesive properties and homogeneity of MPM over PRF.

However, several clinical investigators have recently proposed the use of PRF and MPM membranous forms as substitutes for commercially available barrier membranes in the clinical setting [51, 52]. To our knowledge, there is no published evidence demonstrating that a PRF or MPM membrane can maintain space for tissue regeneration for sufficient periods of time because of their rapid degradation. However, in this study, the most striking histological observation was the formation of a well-formed thick periosteum under the collagen membrane in all MPM tissue sections after 1 month. On the other hand, in PRF specimens, the periosteum started forming in some sections after 8-weeks and became thick and well-formed by 12-weeks. It is possible that in the present study, the increased cross-linking density among the individual fibrin fibers within MPM prolonged the preservation of the MPM membrane at the implantation site, allowing it to serve as a more clinically optimal GBR membrane than PRF.

The mean percentage of bone surface area represents the most important parameter, as it reflects the quality of the newly formed bone. The primary aim of ridge augmentation procedures is to prepare the tissues to receive oral implants; therefore, the success of an implant is related to the quality of the hosting bone. Thus, in line with our histologic findings, the histomorphometric results revealed that MPM boosts bone regeneration faster than PRF does. As noted, PRF reached the first recorded mean percentage of bone surface area in the MPM group after 12-weeks rather than at 4-weeks, like MPM. This booster healing effect of MPM was thought to help in the space maintenance of the defect. Furthermore, this effect helped to overcome the drawback of collagen membrane in which the membrane loses its barrier function within 2 or 3 months, which may not provide sufficient time for completion of the bone regeneration process. It should be clarified that the histologic results obtained in the present study regarding the healing efficiency of MPM and PRF indicate that although both materials promoted healing, MPM was always steps ahead of PRF and showed high osteoinductive potential.

Woven bone made of unmineralized matrix is formed mainly of type I collagen. The replacement of collagenous mesenchymal tissue by mineralization is an indication of bone maturation. Thus, upon bone maturation, there is a decrease in the total collagen percentage in the developed lamellar bone as compared with immature woven bone. In our study, we observed a significant difference in the collagen percentage between MPM and PRF during the first 4-weeks postoperatively. However, no significant difference was found at either 8 or 12-weeks. This indicates the superior quality of the formed mature bone in the MPM-treated group as compared with the PRF-treated group.

Bone regeneration especially after drilling initially involves a typical inflammatory response consists mainly of a leukocyte-rich cell infiltrate and macrophages. These cells are responsible for the secretion of OPN which binds to the bone wound margins contributes to cement line formation. When this process continues, subsequent additions of OPN to the cement lines occurs from osteoblasts differentiating at the wound site. Such OPN deposition is strongly linked to effective integration of the newly formed bone to the preexisting old bone margins found at the site of the drill. This action requires activation of cell signaling leading to extracellular matrix deposition and mineralization [23].

As aforementioned, we were interested in studying the OPN immune profile for both MPM and PRF. Our results were in line with the expression profile of OPN, revealing a significant increase in the OPN mean area percentage in the MPM-treated group during 8-weeks time point as compared with PRF. Furthermore, a uniform linear deposition of the OPN immunoreaction across the cement lines in the MPM group as compared with the less homogeneously dispersed OPN expression throughout the osteon in the PRF group. This indicates that MPM might enhance the production of OPN responsible for an effective homogenous new bone formation and subsequent mineralization.

Based on the results of this study after analyzing bone regeneration parameters, we showed that MPM has a superior effect on fostering the regenerative process in GBR procedures over PRF. Furthermore, our histologic analysis demonstrated the potential osteoinductive nature of MPM over a shorter period, which resulted in a more mature bone with superior quality.

## Conclusion

As aforementioned, we can conclude that MPM could be an excellent choice for the treatment of bone loss in the esthetic zone because of its ability to mold harder and mature compact bone. Moreover, this characteristic offers primary stability and predictability of early implant placement. Both MPM and PRF possess the high bone regenerative capacity and are effective in alveolar bone augmentation. However, MPM has a superior and effective booster healing action than PRF. Furthermore, the rapid bone formation obtained by the MPM group could be advantageous in decreasing the time elapsed between bone augmentation and implant installation in human clinical cases.

## Data Availability

All data included in this current study are available from the corresponding author upon request.

## Declarations

### ARRIVE guidelines

This study has been carried out in compliance with the ARRIVE guidelines for the reporting of in-vivo experiments in animal research. The ARRIVE checklist can be found in the supplementary information.
